# Supplementary material for: Integrated Multi-Tissue Transcriptome Profiling Characterizes the Genetic Basis and Biomarkers Affecting Reproduction in Sheep (Ovis aries)
Source: Genes (Basel). 2023 Sep 27;14(10):1881. doi: 10.3390/genes14101881 (PMC10606288; doi:10.3390/genes14101881)
Supplement: Supplementary file 1 [file genes-14-01881-s001.zip › Supplyment-R1.pdf]

Figure S1: Tissue high variation gene expression heatmap

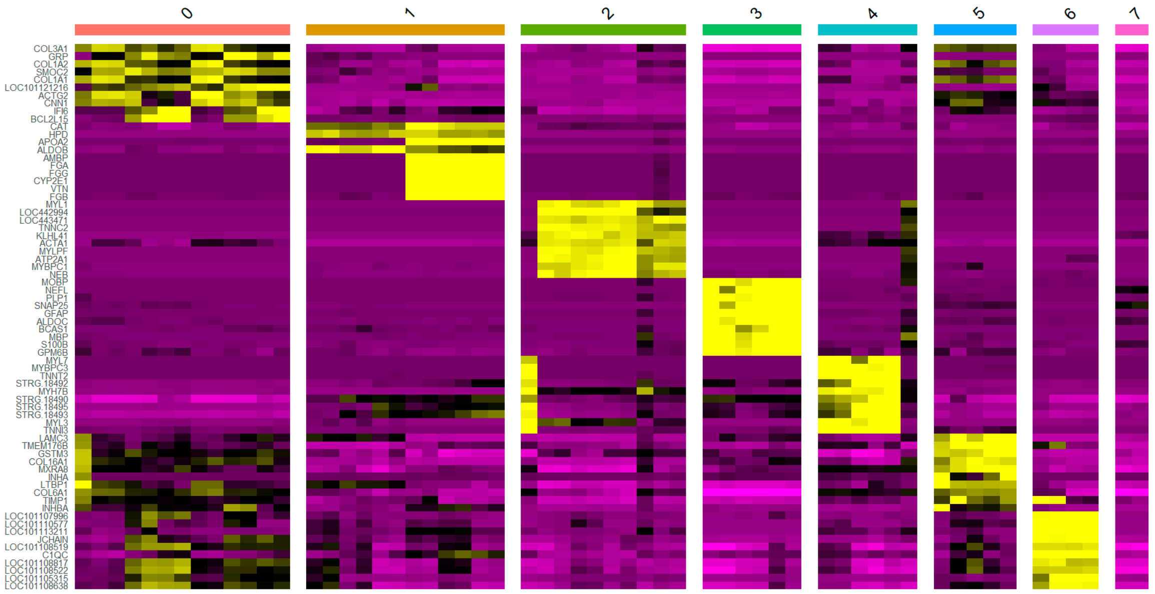

Note: The top10 genes expressed in each cluster

Table S1. Statistics of tissues transcriptome filtered data

Sample	Raw Reads (bp)	Clean Reads (bp)	Clean reads Q30 (%)	GC Content （%）	Overall alignment rates (%)
B1_E027	47,813,270	46,676,488	93.1	49.69	88.24
B2_E027	48,266,892	47,218,604	92.77	49.08	88.55
P1_E027	45,166,074	44,738,134	93.15	49.57	93.1
P2_E027	46,942,856	46,624,700	92.91	47.64	93.65
H1_E027	48,559,696	48,298,886	93.65	41.06	96.33
H2_E027	49,015,056	48,455,294	93.84	41.57	96.12
L1_E027	47,502,950	46,481,884	92.66	43.69	93.51
L2_E027	46,281,274	45,472,572	93.13	43.54	94.5
S1_E027	48,433,418	47,382,068	93.76	51.62	89.19
S2_E027	41,683,908	40,718,050	93.04	50.86	90.78
K1_E027	48,782,722	48,297,090	94.47	47.52	90.47
K2_E027	45,460,410	43,190,974	93.19	43.34	92.95
O1_E027	44,054,012	43,423,122	92.86	51.46	88.24
O2_E027	49,329,374	48,649,988	92.88	51.45	88.65
UH1_E027	45,213,758	43,752,908	93.8	51.06	88.87
UH2_E027	46,730,476	45,822,186	94.35	51.86	88.88

UB1_E027	50,485,840	49,198,276	92.91	49.93	88.33
UB2_E027	46,656,778	45,729,306	93.05	50.73	88.42
M1_E027	46,395,604	46,032,878	93.07	51.68	90.96
M2_E027	42,015,534	41,658,380	92.88	50.62	91.84
B1_B123	49,235,500	47,844,310	92.5	48.39	89.38
B2_B123	47,018,660	46,024,634	92.66	45.67	90.13
P1_B123	45,995,920	45,479,688	92.59	49.55	87.27
P2_B123	42,456,968	41,978,452	92.51	49.38	90.02
H1_B123	53,986,326	53,038,074	93.1	45.43	92.03
H2_B123	49,771,134	48,889,956	92.94	44.22	93.13
L1_B123	45,056,832	43,963,062	92.49	46.78	91.1
L2_B123	45,810,870	44,626,182	92.91	45.63	92.78
S1_B123	41,805,282	40,587,498	92.65	50.91	88.34
S2_B123	46,870,532	45,456,730	93.54	52.72	84.74
K1_B123	49,836,620	48,952,170	93.52	48.45	91.4
K2_B123	48,962,818	47,850,864	93.06	50.09	89.75
O1_B123	47,218,348	46,780,792	93.15	48.95	91.09
O2_B123	48,728,450	47,421,956	93.14	51.01	89.14
UH1_B123	51,040,520	50,174,442	94.82	50.92	90.76
UH2_B123	45,415,458	44,593,402	94.32	52.92	89.19
UB1_B123	44,947,290	43,665,844	93.3	51.8	89.59
UB2_B123	47,139,628	46,264,848	92.98	50.7	90.13
M1_B123	45,807,796	45,271,574	93.29	53.38	89.97
M2_B123	42,599,654	42,205,406	92.64	52.48	90.43
B1_B344	46,253,950	45,068,340	92.53	51.16	86.68
B2_B344	50,075,880	48,966,028	92.39	49.37	88.05
H1_B344	52,999,346	52,272,982	92.94	41.27	95.55
H2_B344	47,482,508	46,754,922	92.62	42.02	96.4
L1_B344	42,019,282	40,965,158	92.19	45.67	91.11
L2_B344	49,445,760	48,385,882	92.67	44.58	91.22
S1_B344	45,848,654	44,729,206	92.78	52.58	82.66
S2_B344	41,589,072	41,044,318	92.18	44.37	83.43
K1_B344	45,934,808	44,691,382	92.6	52.78	92.7
K2_B344	41,131,236	40,617,076	92.15	47.62	89.62

O1_B344	51,361,712	50,674,238	92.6	50.72	88.71
O2_B344	52,852,558	51,977,136	92.67	50.88	87.36
UH1_B344	51,537,420	50,460,510	94.81	52.35	88.14
UH2_B344	50,562,918	49,455,166	94.39	52.57	88.14
UB1_B344	43,081,676	42,251,848	92.93	50.75	88.93
UB2_B344	45,622,124	44,819,352	93.44	49.73	88.88
M1_B344	47,579,894	46,711,884	92.38	50.93	90.16
M2_B344	44,398,236	43,933,442	93.07	51.74	89.89
